# Intratumoral co-activation of ERV transcription and RNA virus-sensing is associated with radiotherapy-induced immune response and abscopal effect

**DOI:** 10.1038/s41698-026-01715-4

**Published:** 2026-10-13

**Authors:** Stella Logotheti, Elif Yıldız, Sarah Hasan, Viktoriia Bulanova, Elpida Theodoridou, Stefan Kuhn, Thorsten Stiewe, Stephan Marquardt, Athanasia Pavlopoulou, Joao Seco

**Affiliations:** 1 Biomedical Physics in Radiation Oncology, German Cancer Research Center (DKFZ), Heidelberg, Germany; 2 Izmir Biomedicine and Genome Center, Izmir, Turkey; 3 Izmir International Biomedicine and Genome Institute, Dokuz Eylül University, Izmir, Turkey; 4 Department of Physics and Astronomy, Heidelberg University, Heidelberg, Germany; 5 Institute of Computer Science, University of Tartu, Tartu, Estonia; 6 Institute of Molecular Oncology, Philipps-University, Marburg, Germany; 7 German Center for Lung Research (DZL), Universities of Giessen and Marburg Lung Center (UGMLC), Marburg, Germany; 8 Genomics Core Facility, Philipps-University, Marburg, Germany; 9 Institute for Lung Health (ILH), Justus Liebig University, Giessen, Germany; 10 Institute of Translational Medicine for Health Care Systems, Medical School Berlin, Hochschule Für Gesundheit Und Medizin, Berlin, Germany

**Keywords:** Biomarkers, Cancer, Computational biology and bioinformatics, Immunology, Oncology

## Abstract

Radiotherapy (RT) transforms tumors into in situ vaccines that trigger antitumor immunity. However, the relevant pathways remain largely unelucidated, limiting clinical translation. Herein, we developed a hybrid dry-lab/wet-lab approach integrating systems-level immune phenotypes following homogenous or spatially fractionated RT (SFRT) with transcriptomic profiling of irradiated tumors. By combining feature extraction with machine-learning, including multilayer perceptron modeling, we ranked predictors of immune infiltration and patient survivability for each RT type. RT induced coordinated upregulation of viral RNA-binding proteins (OASes and RIG I-like receptors) along with endogenous retrovirus (ERV) RNAs predominately 400–800 bp long, which may serve as their ligands. During abscopal responses, OASes and ERV transcripts were co-induced and shared between irradiated and distant tumors. SFRT triggered earlier activation of OAS signaling versus homogenous RT. Our findings highlight ERVs as underappreciated contributors to RT-induced antitumor immunity and their interactions with intratumoral cytosolic sensors as potential novel biomarkers in precision radio-oncology.

## Introduction

Radiotherapy (RT) is employed in the clinic for the management of more than 50% of the real-world oncological patients. Beyond its traditional cytotoxic properties, RT transforms tumors into immunogenic tissues, which train the immune system to recognize and eradicate cancer cells in the body. In rare clinical cases, tumors outside the irradiated area shrink in response to RT. This enigmatic phenomenon, known as “abscopal effect”^1^, has re-invented RT as a powerful means to enhance the efficacy of immunotherapeutics, because its combination with immune checkpoint inhibitors is envisaged to overcome resistance to immunotherapy and increase the frequency of abscopal effects^1^. The immunogenic properties of RT vary depending on the irradiation type^2^. Unveiling the signaling cascades through which RT transforms tumors to in situ vaccines could guide the design of personalized regimens that support durable antitumor immunity at the organismal level.

The immunogenic properties of RT are linked with a cellular stress known as immunogenic cell death^3^. RT induces DNA damage response and repair (DDR/R), leading to cell cycle arrest and death. Dying cancer cells, in turn, release a cocktail of bioactive molecules, including but not limited to, Damage-Associated Molecular Patterns (DAMPs), that reprogram immune cells to elicit antitumor responses^3^. Once the innate immune system senses DAMP motifs through pattern recognition receptors (PRRs), it promotes a pro-inflammatory milieu and sterile inflammation. DAMPs further activate cytotoxic T lymphocytes, immunological memory and adaptive immune responses^3^. A renowned pathway bridging RT-induced DNA damage with immune responses is cGAS/STING. Cytoplasmic double strand DNA (dsDNA) fragments are sensed by the cyclic GMP-AMP synthase (cGAS) which transforms ATP to 2,3-cyclicGMP-AMP (cGAMP). cGAMP activates the stimulator of interferon genes (STING). STING subsequently recruits TBK1 (TANK-binding kinase 1), which then phosphorylates the transcription factor IRF3 (interferon responsive factor 3). The activated IRF3 drives the transcription of a set of immunomodulatory genes, leading to interferon (IFN) secretion and immune response^4^.

Nevertheless, RT-induced immunogenicity does not rely solely on this pathway. Cases have been reported in which the response occurs independently of cGAS/STING, highlighting the co-involvement of additional, still underappreciated pathways. For example, fractionated low-dose irradiation induces a delayed type I IFN response without the involvement of IFN expression or STING signaling^5,6^. Non-conventional activation of interferon-stimulated genes (ISGs) may also occur in response to dsRNA^7^. For example, RT enhances radiosensitization and/or intratumoral lymphocyte infiltration in vivo via activation of a dsRNA-triggered MDA5/MAVS/TBK1 pathway^8^. Consistently, RT stimulates the autonomous release of dsRNA from cancer cells to activate TLR3-mediated IFN-I signatures that facilitate antitumor immune responses^9^. The dsRNA triggers of these alternative pathways may be transposable elements (TEs), mobile repetitive nucleic acid sequences that have been incorporated into the genome through spontaneous integration, accounting for almost 50% of human DNA. Pan and colleagues have reported that epigenetic de-suppression of endogenous retroviruses (ERVs), a subclass of TEs, triggered MDA5/MAVS-mediated type I interferon signaling leading to radiosensitization and antitumor immunity^10^. TEs reside in the so-called “junk DNA” regions and are surfacing as instructors of the immune system^11^. Although they are no longer mobile, a subset of them can be mobilized and transcribed in cancer cells. Activation of these elements by RT generates a reservoir of immunogenic RNA species capable of eliciting IFN cascades^11^. This novel non-coding RNA arm of RT-induced immunogenicity remains largely enigmatic, compared to the well-characterized cGAS/STING.

Intriguingly, when RT is delivered to a tumor heterogeneously, for example, in spatial fractions (spatially fractionated RT, SFRT), it elicits stronger anti-tumor responses than the conventional, homogenous RT. In colorectal cancer mouse models, simultaneous administration of high-dose to one half of the tumor and low-dose to the other half, generated a more reactive tumor immune microenvironment compared to the homogenously irradiated controls^12^. Other studies used microbeam (MRT) or minibeam (MBRT), where a collimator delivers extremely high-dose-rates into high-dose areas (‘peaks’), separated from low-dose regions (‘valleys’) by a few hundred micrometers or millimeters, correspondingly^13^. In preclinical rodent models, this type of spatial fractionation consistently induced superior in vivo antitumor immunity phenotypes along with fewer side effects^14–16^. Most recently, MBRT achieved complete clinical response and abscopal effects in a patient with recurrent metastatic sinonasal melanoma who had been unresponsive to conventional X-rays combined with checkpoint inhibitors (CPIs)^17^. Another case of advanced melanoma has been reported, where the patient showed complete response to a combination of SFRT and CPIs and remains disease-free for 2.5 years^18^. Collectively, these findings highlight a novel paradigm, in which heterogeneous intratumor irradiation represents an appealing immunotherapy partner, due to its reduced toxicity and increased immunogenicity^19^.

The irradiated tumor undergoes transcriptional changes that depend on the type and/or dose of RT^2^. The transcriptional programs that are activated within the tumor to orchestrate the in situ vaccine effects of different RT types, doses and delivery modes remain uncovered, but they could illuminate the relevant mechanisms. Herein, we leverage studies on murine cancer models in which homogeneous or heterogeneous RT elicited immunogenic responses at an organismal level. We associate the phenotypes of RT-induced anti-tumor immunity and abscopal effects with transcriptional changes in both protein-coding genes and TEs within the targeted tumors. Our study unveiled simultaneous co-transcription of ERVs and viral RNA sensors induced within tumor cells by conventional X-rays and SFRT and highlighted key differences in terms of immunogenic response to RT and survivability prediction in patients.

## Results

### Broad antitumor immunity phenotypes are associated with tumor transcriptional reprogramming in response to conventional RT and SFRT

We mined for all the publicly available studies where rodent tumor models received any type of RT and demonstrated robust phenotypes of local and/or systemic anti-tumor immune responses. Eight studies across six cancer types with their corresponding bulk transcriptomics data (RNA-Seq or RNA microarrays) were eligible^14,16,20–25^ (Table 1), whereby conventional X-ray irradiation (CONV) was applied in a total dose range of 8–30 Gy. In the lymphoma study^24^, one sequenced tumor was located within the RT-targeted field and the other one outside the field. This setting allowed us to detect DEGs in non-irradiated (NIR) tumors that represent reporters of abscopal effects. In two studies (^14^ and GSE56113), CONV was compared to microbeam RT (hereafter referred to as SFRT), providing insights into transcriptional reprogramming associated with the in situ vaccine effects of each dose delivery modality. For each dataset, transcriptomic analyses identified up- and down-regulated transcripts (log2FC ≤ −1.5 and ≥1.5; *p* value ≤ 0.05) in treated versus untreated controls.

**Table 1:** Overview of studies where mouse phenotypes of in situ vaccine effects have publicly available transcriptomics data of the ‘immunogenic‘ targeted tumors

Study accession number	Cancer type	Model	Irradiation Type	Transcriptomics platform	Time of sample collection post-RT	Sample undergoing RNA-Seq (irradiated vs. non-irradiated controls)
GSE207717	Pancreatic adenocarcinoma	KPC syngeneic mouse (strain C57BL/6J) and PDAC organoids derived from KPC-OG mice (KPOG)	X-rays, 1 fraction of 8 Gy	Illumina NovaSeq 6000 (Mus musculus)	24 and 48 h post irradiation	KPOG organoids
GSE281695	Lymphoma	BALB/c mice with two A20 lymphoma tumors	X-rays, 1fraction of 8 Gy or 2 fractions of 4 Gy	Illumina NovaSeq X Plus (Mus musculus)	24 h and 7 days post treatment.	Tumor inside and outside the irradiated area
GSE168016	Colorectal	MC38 syngeneic mice (strain C57BL/6J)	X-rays, 1 fraction of 15 Gy	Illumina NovaSeq 6000 (Mus musculus)	60 h	tumor cells
GSE209765	Colon	CT26 syngeneic mice (BALB/c or C57BL/6)	X rays, 1 fraction of 8 Gy	DNBSEQ-G400 (Mus musculus)	When tumor reached 2000 mm^3^	Tumor treated with mock immunotherapy and received RT two days later
GSE268311	Melanoma	B16 melanoma mice (strain C57BL/6)	SFRT (microbeams) vs. X-rays	NanoString nCounter® XT Assay	2 days and 7 days post-RT. Non-irradiated controls collected 12 days post-tumor implantation.	Tumors
GSE56113	Glioblastoma	orthotopic rats bearing intracranial 9 L brain tumors	SFRT (microbeams) vs. X-rays	Affymetrix GeneChip® Rat 230_ 2	6 h, 48 h, 8 days and 15 days after treatment	tumors
GSE61208	Breast	4T1 syngeneic mice	X-rays, 5 ×6 Gy	[Mouse430_2] Affymetrix Mouse Genome 430 2.0 Array	4 days post-RT	Tumors
GSE255242	Breast	Murine transplantable TS/A tumor-cell-line co-engrafted with CAFs in syngeneic mice	X-rays, 2 ×6 Gy	NextSeq 2000 (Mus musculus)	when the tumor size reached 2000 mm^3^.	Tumor treated with RT when reached a volume of 80–100 mm³

**Fig. 1:**
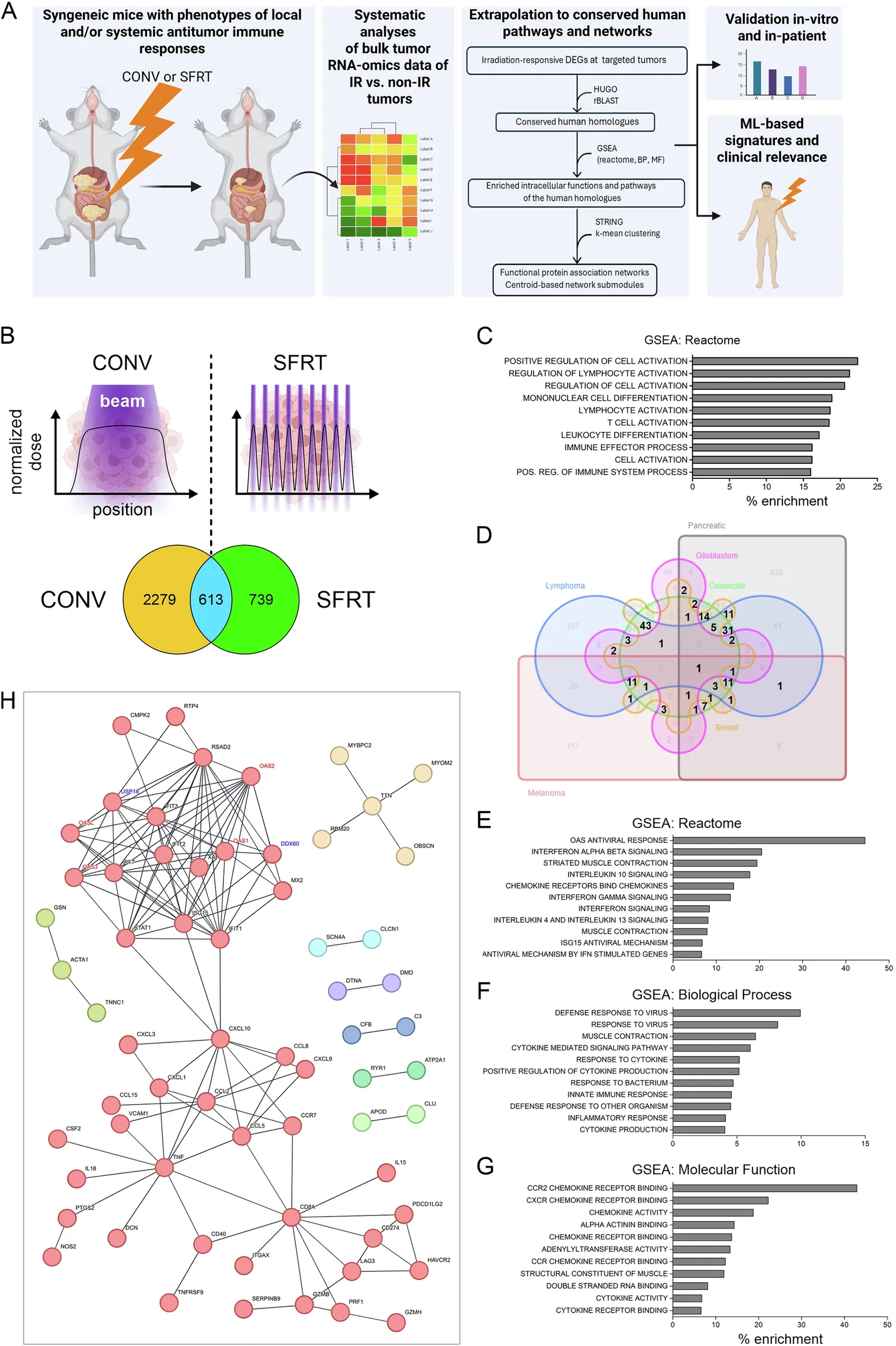
Association of antitumor immunity/abscopal phenotypes with transcriptional changes in response to CONV RT vs. SFRT. **A** Workflow of the systemic meta-analysis (created with Biorender). RNA-Seq data from irradiated tumors extracted from mice with phenotypes of antitumor immune response were compared to their non-irradiated controls. The human orthologues of upregulated genes were defined and subjected to functional enrichment and network analysis, as well as machine learning-facilitated correlations with clinical patient data. **B** Venn diagram of conserved genes upregulated by SFRT vs. CONV RT. **C** GSEA reactome analysis of the 613 genes shared between CONV and SFRT. **D** Venn diagram of CONV-RT upregulated gene transcripts per cancer type. Genes common across ≥ 3 cancer types are denoted with a number, with a total number of 161. **E-G** GSEA for reactome (**E**), biological process (**F**) and molecular function (**G**) of the 161 shared genes, **H** STRING network reconstruction of the interactions among the protein products of the 161 genes. The submodule of 46 red hubs are associated with IFN signaling and response to viruses, including OAS proteins, ISGylation proteins, and ISGs. The light-yellow submodule contains 5 proteins highly specific for striated muscle contraction.

To extrapolate the transcriptional changes related to the mouse anti-tumor immunity phenotypes to humans, we identified the human orthologues of the RT-upregulated transcripts and performed downstream functional in silico analyses according to the pipeline described in Fig. 1A. Most upregulated rodent genes have human counterparts, while the small percentage of non-conserved genes may account for rodent-specific responses (Supplementary Fig. 1A). We then pooled the human homologues of all CONV-upregulated (across six cancer types) and the SFRT-upregulated transcripts (across two cancer types). We found a substantial overlap of genes transactivated by both RT types (Fig. 1B), which are associated with the activation and/or regulation of several immune cells, such as lymphocytes, T cells and mononuclear cells (Fig. 1C). There were also genes specific for each RT modality. Hence, we postulated that some of these genes may account for the reported differential effects of SFRT versus CONV and proceeded to analyze the transcriptional responses for each modality separately.

### Conventional radiotherapy activates IFN-dependent RNA viral immune responses at the irradiated tumor sites

Analysis of CONV-treated tumors versus their untreated controls across the six types of cancers (Table 1) identified 161 genes that were commonly upregulated in ≥3 of the 6 cancer types (Fig. 1D). GSEA of their human homologues revealed associated intratumoral pathways and processes (Fig. 1E). As expected^26^, the upregulated transcripts were associated with IFN cascades. Viral responses mediated by the 2′-5′ oligoadenylate synthase (OAS) proteins, that particularly sense cytosolic viral dsRNA^27^, were remarkably enriched. The IFN-dependent ISG15 pathway, which closes the cell gates to DNA and RNA viruses by marking them through a post-translational modification^28^, was also enriched. Biological processes were associated with response to viruses, cytokine production and signaling, and activation of innate immunity and inflammatory responses (Fig. 1F), while molecular functions highlighted double-stranded RNA binding and adenyltransferase activity (Fig. 1G). These findings suggest enhanced sensing and response to cytosolic dsRNA following CONV RT. We then reconstructed the physical and functional associations among the protein products of the 161 genes using STRING, to explore protein-protein interactions. K-means clustering revealed a highly interconnected network of 64 proteins, with a prominent submodule (46 hubs) related to IFN signaling and response to viruses. This submodule entails the entire family of OAS proteins (OAS1, OAS2, OAS3, OASL), key components of the ISG15 machinery (ISG15 and USP18), STAT1, and ISGs which combat RNA and DNA viruses (Fig. 1H). These findings associate immunogenic CONV RT doses with transactivation of IFN-dependent pathways of viral immunity in the irradiated tumor, with a particular preference for cytosolic sensors of RNA viruses.

**Fig. 2:**
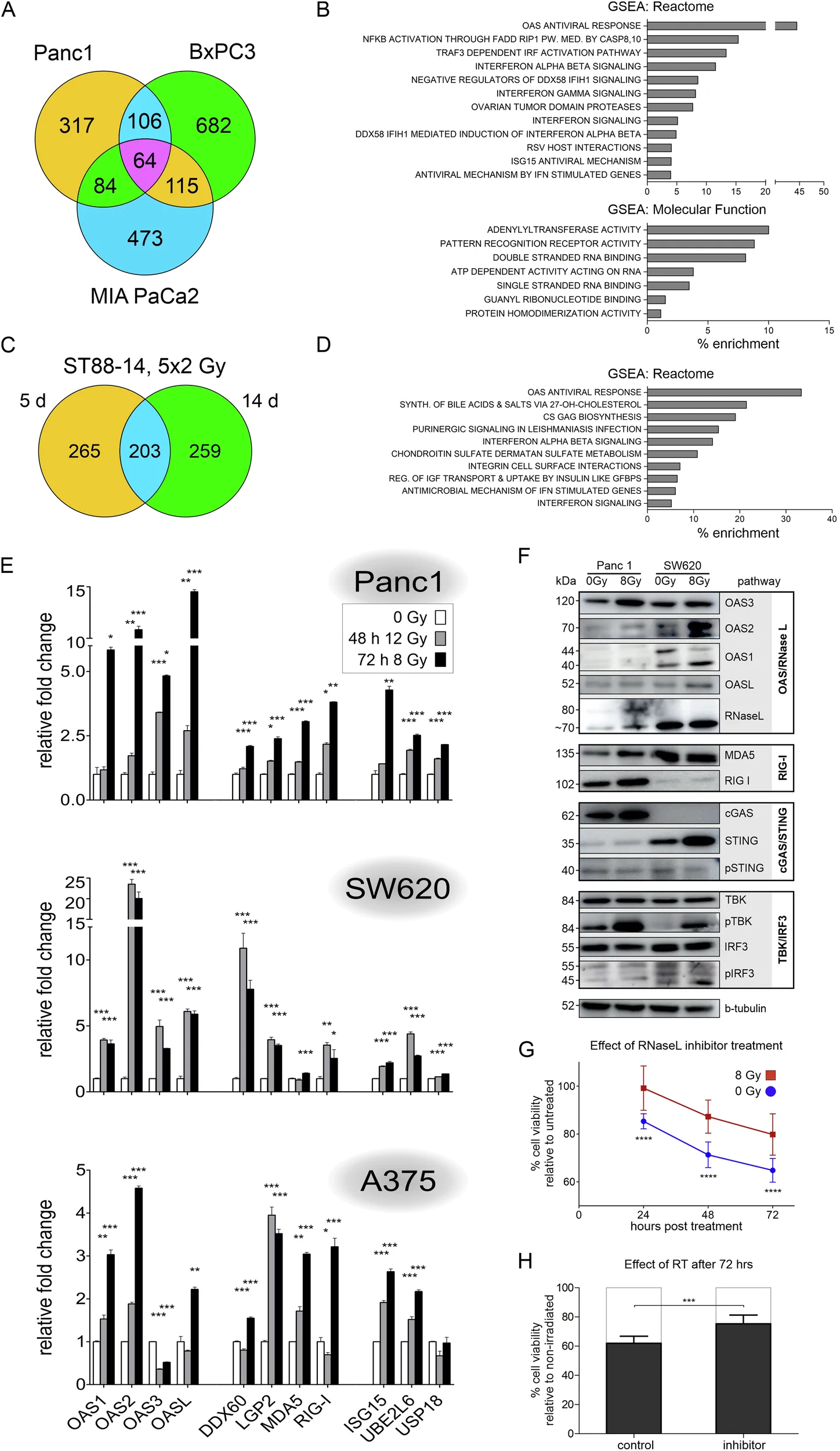
Activation of innate immune responses to RNA viruses within tumor cells treated with CONV RT. **A** Venn diagram of upregulated gene transcripts in Panc1, BxPC3, and MIA PaCa2 cell lines undergoing ICD 72 h after CONV-RT (3×8 Gy) reveals 64 shared genes **B** GSEA of reactome of the 64 genes, **C** Venn diagram of upregulated gene transcripts in ST88-14 cells 5 and 14 days after treatment with a 5x2Gy regimen. **D** GSEA of reactome of the 64 genes in the transcripts that remain upregulated between 5-14 days post-RT. **E** qPCR for components of the OAS/RIG-I system and ISG15 pathway in irradiated Panc1 (pancreatic), SW620 (colorectal) and A375 (melanoma) cell lines 48 h after 12 Gy and 72 h after 8 Gy. RNA levels are presented relative to corresponding levels in non-irradiated cancer cell lines (0 Gys) after 72 h. Statistically significant differences with *p* value ≤ 0.05 (*), ≤ 0.01 (**) and ≤ 0.001 (***) are denoted. **F** Western blots for monitoring of changes in the protein levels of the components of OAS/RNAseL, RIG I and cGAs/STING pathways, 72 h post-rt in Panc1 and SW620 cell lines. **G** Cell survival assay in non-irradiated versus irradiated Panc1 cells treated compound17a, which binds to the ATP-binding pocket of RNase L and inhibits ribosomal RNA cleavage. Survival was estimated at 24, 48 and 72 h after treatment. The values are normalized to the corresponding DMSO-treated controls. **H** Percentage survival change in response to irradiation, in RNAse L inhibitor-treated versus DMSO-treated Panc1 cells 72 h after treatment.

### OAS/RNAseL and RIG-I signaling pathways are transactivated within tumor cells after treatment with immunogenic regimens of conventional radiotherapy

Since tumors are heterogeneous tissues, the identified pathways might derive from cellular components other than tumor cells, such as immune cells or fibroblasts. To clarify if these pathways are induced within the tumor cells, we analyzed two independent datasets of irradiated human cancer cell monocultures of pancreatic adenocarcinoma cells (PDAC, ArrayExpress, ref. no. E-MTAB-13096)^4^ or soft tissue sarcoma cells (malignant peripheral nerve sheath tumor)^29^. By analyzing the corresponding transcriptomes of the PDAC cell lines (Panc1, BxPC3 and MiaPaCa2) that exhibit immunogenic cell death upon RT, we found 64 significantly upregulated genes shared across the three irradiated PDAC cell lines 72 h post-treatment (Fig. 2A). These genes were enriched for the IFN-dependent viral innate immunity pathways OAS, RIG-I/MDA5, and ISG15 (Fig. 2B), as well as pattern recognition receptors, adenylase activity, and RNA binding (Fig. 2C). Similarly, the peripheral nerve sheath tumor cell line ST88-14 showed a persistent activation of a set of transcripts significally associated with OAS and IFN responses, 5-14 days post-treatment with a fractionated regimen 5 x 2 Gy (Fig. 2D). These data demonstrate that transactivation of the RNA viral sensors occurs within the irradiated tumor cells.

To experimentally validate the in-silico findings, we irradiated human cell lines from three different cancer types (colon, pancreatic and melanoma) and isolated RNA after 72 h and 48 h exposure to 1x8 Gy and 1x12 Gy, correspondingly. Then, we performed qPCR to monitor the transcriptional changes of PRRs that are involved in the recognition of viruses by the innate immune system, including OAS family members, retinoic acid-inducible gene-I-like receptors (RLR) and the components of the ISG15 pathway. All candidates were actively transcribed within the RT-treated tumor cell monocultures versus the non-irradiated controls (Fig. 2E). For most players, induction was robust at 8 Gy/72 h, implying that adequate time after irradiation may be required for the establishment of these transcriptional programs. In pancreatic (Panc1) and colorectal (SW620) cells, which showed profound fold changes in RNA PRRs, we further estimated the protein levels of OASes, RLRs and their downstream effectors (Fig. 2F). OAS1-3 are active enzymes that oligomerize ATP to 2′-5′-oligoadenylate, which subsequently binds to RNase L. The activated RNAse L cleaves dsRNA and ssRNA and the shorter fragments are used as substrates of the RLRs MDA5 and RIG-I. The fourth OAS family member, OASL, lacks catalytic activity but it can physically associate with RIG-I to enhance RIG-I signaling^27^. We found that in both cell lines, OAS2 and OAS3 are upregulated after 72 h in response to 8 Gy compared to the untreated controls. SW620 cells further showed a mild induction of OAS1 and OASL. The levels of RIG-I and MDA5, and their downstream effectors phospho-TBK1 and phospho-IRF3 were also elevated. Interestingly, Panc1 showed higher basal levels of RIG-I, while SW620 showed higher basal MDA5 levels (Fig. 2F). Consistent with previous studies^4^, in irradiated Panc1 cells, cGAS and STING protein levels are enhanced relative to their non-irradiated counterparts. In SW620 cells, RT enhances STING protein levels, but cGAS levels are undetectable (Fig. 2F), in line with work reporting that cGAS is not present in all CRC cell lines^30^. Overall, immunogenic RT doses activate OASes and RIG I/MDA5/TBK/IRF3 pathways within tumor cells.

If OASes are passive markers of IFN I pathway activation, then blocking of the OAS/RNAseL pathway would not affect RT responses. Subsequently, we treated Panc1 cells using compound 17a, a thiophen-4-one that potently inhibits RNAse L activity in cancer cells in doses as low as 2 µM^31^. When Panc1 cells are treated with 4 µM compound 17a, cell survival decreases over time. However, when they are subsequently subjected to 8 Gy RT, survival is increased relative to the non-irradiated counterparts, indicating that RNAse L inhibition antagonizes RT effects (Fig. 2G). At 72 h, cell survival in response to RT is significantly lower in DMSO-treated cells than compound 17a-treated cells (61.93 vs. 75.29%, respectively, *p* value: 2 x 10^−8^), indicating resistance of Panc1 cells to RT-induced cell death upon RNAse L inhibition (Fig. 2H). These findings suggest, at least in part, an active role of the OAS/RNAseL pathway in RT responses.

### SFRT induces OAS antiviral and NK responses earlier than conventional radiotherapy and establishes signatures with higher predictability for longer patient survival

**Fig. 3:**
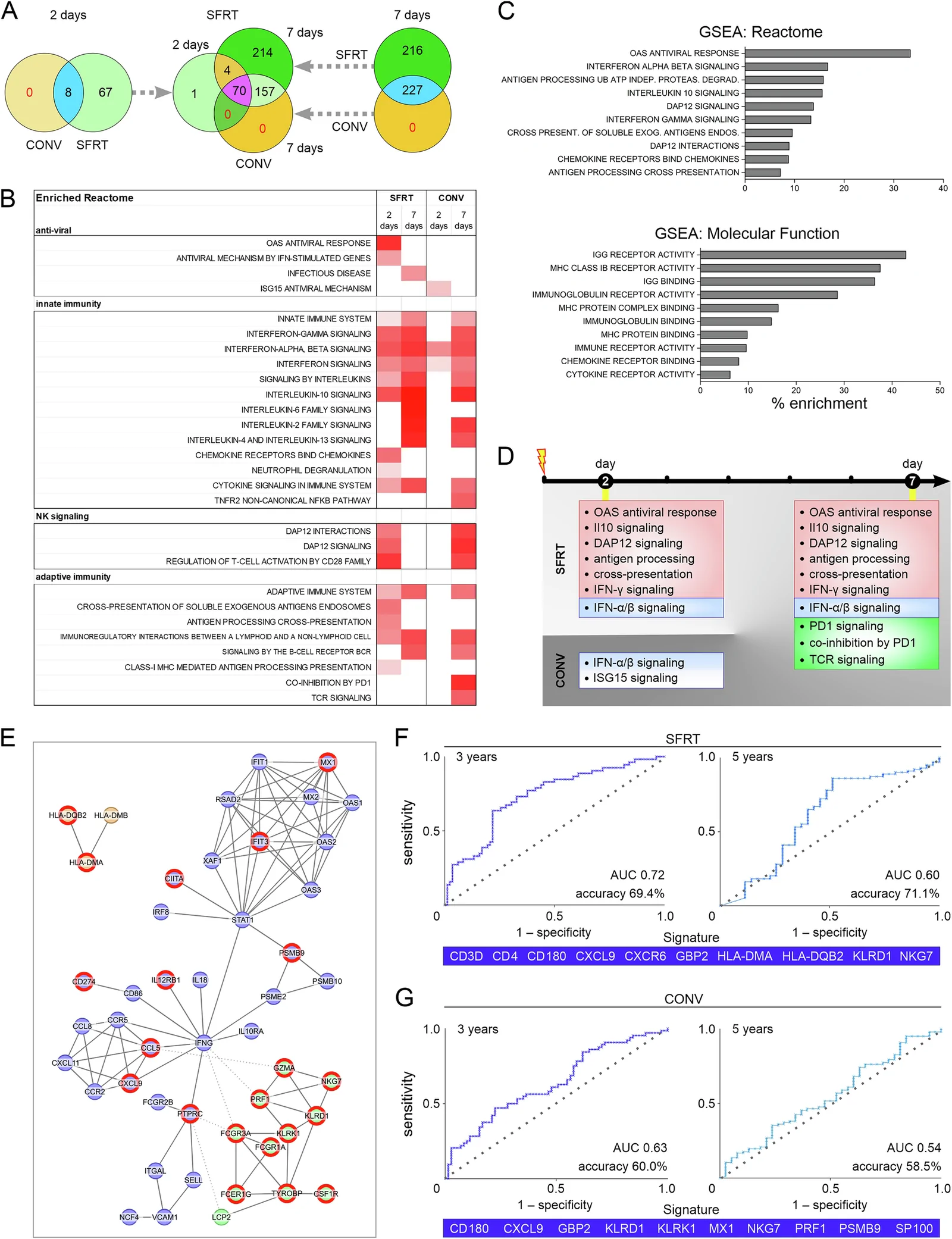
SFRT transactivates OAS antiviral responses earlier and produces signatures of response to SFRT are correlated with immune cell infiltration and better predictability of ≥ 3-year survival in melanoma patients more robustly than CONV RT. **A** Venn diagrams of SFRT or CONV-RT upregulated gene transcripts in melanomas excised at day 2 (left) or day 7 (right) after irradiation treatments. Comparison of SFRT transcriptomes at days 2 and 7 with CONV RT transcriptomes at day 7 unveils 70 shared upregulated genes. **B** heatmaps of overrepresented reactomes 2 and 7 days after SFRT or CONV RT. **C** Heatmaps of overrepresented reactome processes of the 70 common genes. **D** A timeline summary of activation of reactome processes by CONV RT vs. SFRT. **E** STRING analysis of the protein products of the 70 genes. **F** ROC curves correlating the SFRT-induced transcript signature with survival of melanoma patients ≥3 years (left) and ≥ 5 years (right) after initial diagnosis, **G** Same as (**F**) for the CONV RT-induced transcript signature. Survival prediction accuracy is higher in the case of the SFRT signature, for both cut-offs.

ASTRO guidelines conditionally recommend stereotactic body RT for melanoma tumors >5 cm^18^. In Mayo clinic, two heavily-treated patients with bulky metastatic melanomas and no other therapeutic alternatives received SFRT for symptomatic relief, due to the ability of SFRT to cover large tumor surfaces^17,18^. Both patients showed tumor shrinkage, abscopal responses to metastatic sites, re-sensitization to immunotherapy, and are, to date, still alive. In agreement, Trappetti and colleagues demonstrated that in melanoma mouse models, SFRT conferred immunogenic advantages and improved tumor control compared to CONV RT^14^. Due to the ability of SFRT to induce abscopal effects in melanoma at the preclinical and clinical levels, we focused on the corresponding transcriptomics dataset of the Trappetti study (Table 1, GSE268311) to get transcriptional insights on the SFRT-primed immunogenic signaling. In this study, mice were treated with SFRT or CONV RT, and the corresponding tumors were subjected to mRNA microarrays 2 and 7 days post-irradiation, allowing detection of SFRT-responsive transcripts in a time-dependent manner. GSEA of the inferred human homologues revealed upregulation of 76 transcripts at day 2, eight of which are commonly activated by SFRT and CONV, while the remaining are SFRT-specific. By day 7, 216 conserved transcripts were induced by SFRT and 227 by both SFRT and CONV. Notably, 70 of the 76 genes induced by SFRT at day 2 remained actively transcribed in SFRT-treated melanomas and only began to be expressed in the CONV-irradiated ones at day 7 (Fig. 3A). Comparison of the top-enriched pathways following SFRT indicated earlier induction of pathways related to the OAS antiviral response, IL10 and interferon signaling, DAP12 signaling, and antigen processing cross-representation (Fig. 3B). Late SFRT responses are associated with the maintenance of IL10 activity and the induction of additional interleukins, such as IL2, IL6 and IL4/IL13 (Fig. 3B). The CONV RT triggered early activation of ISG15 antiviral responses and IFN signaling, followed by late activation of the PD-1 and TCR signaling pathways at day 7 (Fig. 3B).

The persistently SFRT-upregulated set of 70 DEGs was enriched for OAS antiviral response, ubiquitin-independent proteosomal degradation and DAP12 signaling (Fig. 3C, reactome), along with immunoglobin binding and activity  (Fig. 3C, molecular functions). These processes are enriched since day 2 in the SFRT-treated tumors (Fig. 3D).The corresponding protein products of the 70 genes  form a core network of 47 hubs, related to IFN α/β signaling (blue cluster, 33 hubs), NK cell-mediated toxicity (green cluster, 11 hubs) and peptide antigen assembly with the MHC II protein complex (orange cluster, 3 hubs). The components of the OAS viral response are clustered in a distinct submodule that connects to IFNγ via STATs (Fig. 3E). Similar results were obtained when we analyzed the microarray mRNA data from glioblastomas at 2 or 8 days post SFRT treatment (Table 1, GSE56113) (Supplementary Fig. 2A–C). Since OAS antiviral response was activated earlier, we examined whether OAS gene expression is associated with these cell types in patient melanomas. Analysis using TIMER2.0^32^ revealed that all OAS genes are positively correlated with NK (Supplementary Fig. 2D) and DC (Supplementary Fig. 2E) infiltration. Conversely, melanoma patients with OAS2 mutations exhibited decreased NK infiltration (Supplementary Fig. 2F). Collectively, these results suggest that SFRT induces OAS-mediated RNA antiviral immunity, IFN responses and DAP12 signaling earlier than CONV.

Finally, considering that SFRT prolonged the survival and re-sensitized melanoma patients to immunotherapies^17,18^, we sought to define characteristic transcript signatures induced by SFRT compared to CONV RT type and associate them with prediction of survival outcomes in human melanoma patients. Due to the lack of samples from SFRT-treated patients, we used survival data from the SKCM cohort of the TCGA, as a commonly referenced clinical setting. We hypothesized that if SFRT confers superior disease outcomes relative to CONV RT, then the SFRT-activated transcriptome should correlate more strongly with longer patient survival. To produce manageable signatures from the irradiated mouse transcriptomes, we prioritized the most significant upregulated transcripts, using XGBoost and Random Forest; we identified their human homologues; and we ranked the top 10 homologues for each RT modality by decreasing composite score. SFRT-specific and CONV-specific signatures appear at the bottom of Fig. 3F and Fig. 3G, respectively. Multilayer perceptron modeling estimated the predictive potential of the SFRT- and CONV RT-induced signatures for patient survivability at 1, 3 or 5 years after initial diagnosis. As indicated by the ROC curves (Fig. 3F, G), the SFRT-induced immunogenic signature performed better than the CONV-induced one, especially for the 3-year period cut-off, with 69.4% accuracy (AUC: 0.72) versus 60% accuracy (AUC: 0.63). For the 5-year survival cut-off, the respective values were 71.1% (AUC: 0.60) and 58.5% (AUC: 0.54). Collectively, the SFRT-induced transcriptional signature exhibited better predictive capacity for patient survivability.

### RT-induced intratumoral upregulation of viral RNA cytosolic sensors is accompanied by clinically-relevant changes in the transcription of TE subpopulations

**Fig. 4:**
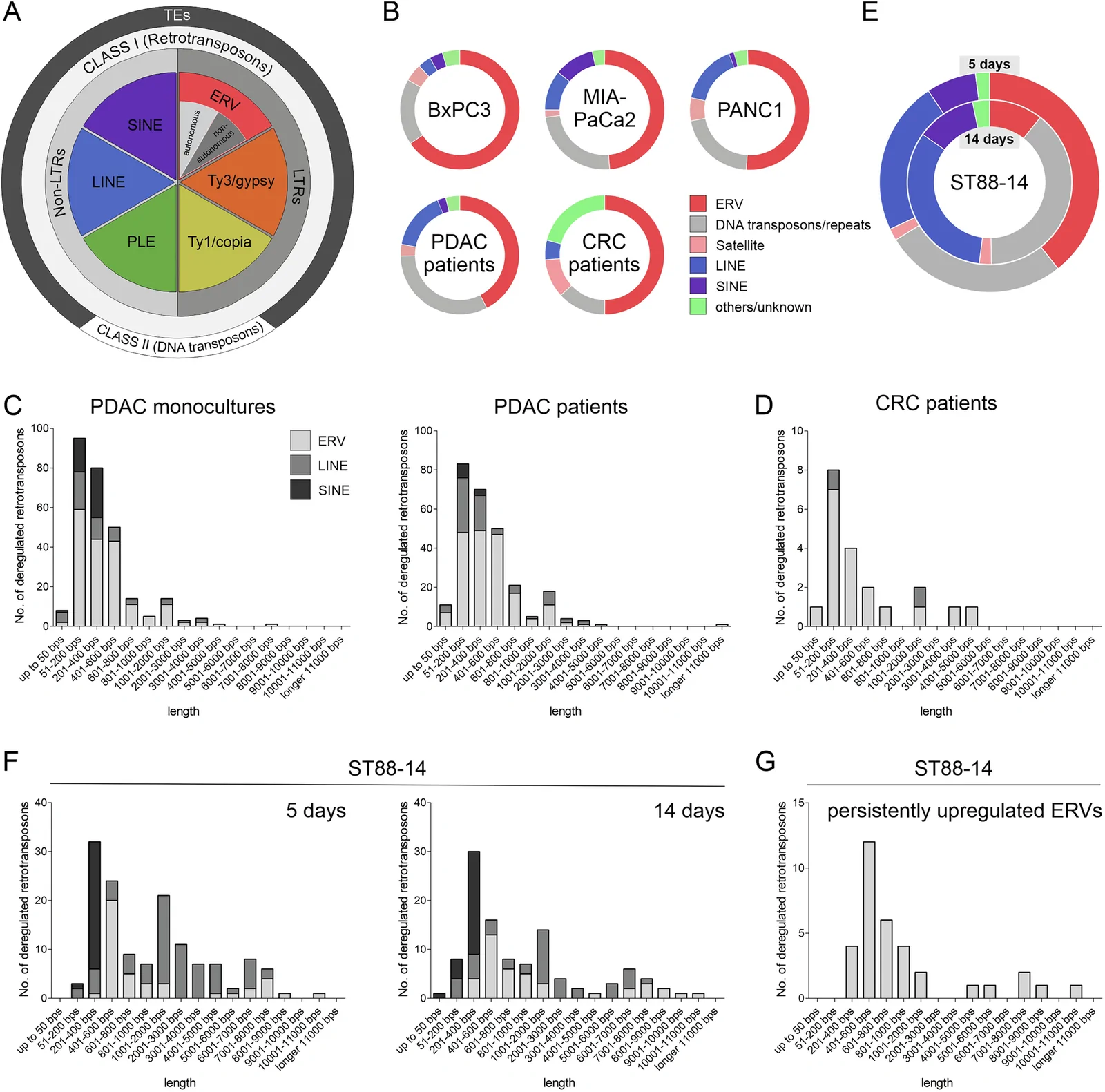
Global TE transcription changes in irradiated human tumor cells. **A** Classification of eucaryotic TEs. Class I (retrotransposons, including LTR/ERVs, LINEs and SINEs) require an RNA intermediate to transpose, and Class II (DNA transposons) move through a DNA intermediate. PLEs Penelope-like elements, LINEs Long interspersed nuclear elements, SINEs Short interspersed nuclear elements (adapted and modified from ref. ^39^). **B** Doughnut plots of the differentially expressed groups of TEs in irradiated vs. non-irradiated PDAC cell lines, PDAC patients and CRC patients. In all cases, the most prominent transcriptional changes are observed for the LTR family (mainly ERVs), followed by upregulation of DNA retroposons. **C** Length distributions of the deregulated retrotransposons in the corresponding irradiated human PDAC cell lines (pooled) and PDAC patient tumors. **D** Same as (**C**) for CRC patient tumors. **E** Doughnut plots of differentially expressed TE groups in irradiated ST88-14 cells, 5 days (outer circle) and 14 days (inner circle) post-RT. **F** Length distributions of the deregulated TE groups in ST88-14 cells at 5 and 14 days post-RT. **G** Length distributions of the ERVs that are commonly upregulated in days 5 and 14.

A characteristic feature of the RNA sensors that were recurrently upregulated upon RT, is that they are inherently unable to recognize dsRNA sequences^33^. Instead, they are specific for the A-form structure of dsRNA, while different sensors are sensitive to distinct length ranges^34^. RIG-I recognizes lengths ~22–500 bp; MDA5 is specific for dsRNA lengths ~500–1000 bps; OAS1 and OAS3 recognize lengths <50 bps and >50 bps, correspondingly, while the specific length recognized by OAS2 remains unknown^35^. Upon dsRNA binding, these sensors undergo conformational changes that trigger downstream type I IFN and NF-kappaB pathways^36^. At least in some cancers, it has been experimentally proven that RIG-I and MDA5 bind TEs, mainly ERVs, to activate antitumor immunity^37,38^. Therefore, we searched whether ERVs and/or other TE classes (Fig. 4A, adapted from^39^) that could act as potential ligands of these sensors are expressed in the irradiated tumors and tumor cell monocultures.

We preferably used TEtranscripts2, a specialized software that incorporates TE-associated reads in the differential expression analysis of human RNA-Seq data, discriminates between Class I (retrotransposons, including LTR/ERVs, LINEs and SINEs) and Class II TEs (DNA transposons), and has outperformed other similar tools^40^. Analysis of RNA-Seq data from the pancreatic cancer cell lines^4^ indicated global TE changes the LTR family (mainly ERVs) exhibited the most frequently deregulated class, accounting for 49–65% of all the differentially transcribed TEs. We evaluated the clinical relevance of this finding in surgically excised tumors from PDAC patients manifesting immune responses following RT compared to matched untreated patients^41^. The pattern of TE alterations in the irradiated tumors was consistent with that of the human PDAC cell monocultures (Fig. 4B). The same pattern was recapitulated in colorectal (CRC) tumors obtained from patients pre- and post-RT who were responsive to immunotherapy^42^ (Fig. 4B).

To infer whether the upregulated TE populations are recognizable by the upregulated dsRNA sensors, we classified them by nucleotide size. We found that the most upregulated TEs are between 50 and 800 bps, a range that corresponds to the duplexes that are sensed by OAS1/3, RIG-I and MDA5. The majority of the 50–800 bp-long TE transcripts are mostly LTR/ERVs, followed by LINEs and SINEs (Fig. 4C). CRCs also show an enhancement of ERVs at the range of 50–200 bps, a length that correlates with peak activity of RIG-I (Fig. 4D). Similarly, in the irradiated ST88-14 monocultures, 5 days after RT, ERVs are deregulated in a percentage of 39.3%, whereas LINES and SINEs represent the 22.6% and 7.3% of the deregulated TEs (Fig. 4E). Upregulated retroelements, including ERVs, LINEs and SINEs show a broad length range at 5 and 14 days post-RT. TEs in the ranges 400–600 bps and 600–800 bps derive mostly from the ERV subclass (Fig. 4F). Although in day 14, the percentage of deregulated ERVs drops to 10.5% (Fig. 4E), most of the upregulated LTR/ERV transcripts (33 of 42 ERVs) at this time point are the same ones that are upregulated at day 5, indicating the persistent activation of a specific ERV set during RT responses. Interestingly, the length of this persistent set ranges between 200 and 1000 bps, with most of them in the range of 400–600 bps (Fig. 4G). Overall, RT induces global changes in intratumoral TE subpopulations, with predominant upregulations of LTR/ERVs in length ranges that can be recognized by the induced RNA sensors.

### Autonomous LTR/ERVs 400–800 bp long are transcribed in immunoreactive mouse and human tumors following RT

**Fig. 5:**
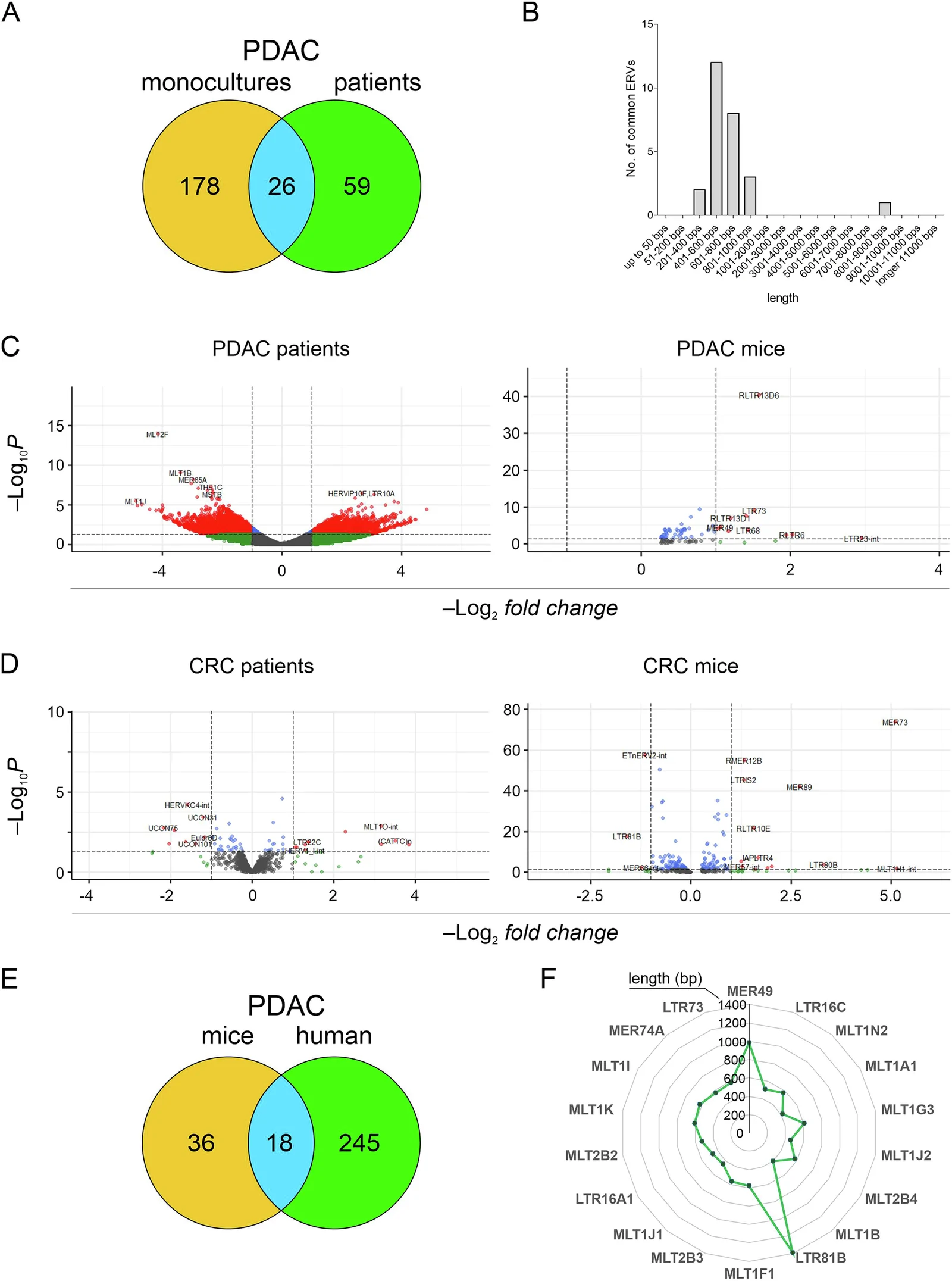
Increased transcription of autonomous ERVs 400–800 bp in length is conserved in mouse and human PDACs that exhibit RT-induced immunogenicity. **A** Venn diagram of commonly upregulated autonomous ERVs in human PDAc cell lines. **B** Length distribution of the clinically relevant 26 upregulated ERVs in PDACs. **C** Volcano plots of upregulated and downregulated LTR/ERV transcripts in irradiated vs. non-irradiated mouse PDACs (left) and CRCs (right); red and green dots indicate up- and down-regulated genes, respectively, while gray and blue dots represent genes without statistically significant changes. The most responsive ERVs are annotated. **D** Venn diagram of the shared upregulated ERVs among irradiated human PDAC cell lines and/or PDAC tumors and mouse PDACs. **E** Length distributions of the conserved 18 upregulated autonomous ERVs. **F** Radar diagram of the length distributions of the conserved upregulated autonomous ERVs. Their identifiers are noted in the outer circle.

Most of the human ERVs are non-autonomous LTRs, meaning that do not encode proteins and primarily act as *cis* regulators. However, a small subset is autonomous, approximating near full-length proviral sequences and retaining the ability to synthesize viral proteins^40^. In autonomous ERVs, LTR sequences flank protein-coding genes that, when translated, act as tumor-associated antigens eliciting T cell-mediated immunological recognition^43^. Due to their potential to synthesize peptides that behave as neoantigens^43^, we wondered if RT induces ERVs with protein-coding potential. We utilized a pipeline, called ERVmap, which is highly suitable for the sensitive quantification of human autonomous ERVs from RNA-Seq data, because it uses a manually curated, annotated database of 3.220 autonomous ERVs^40^. As the original ERVmap script was written in Perl, a language now rarely used, we re-implemented the pipeline in the most popular Python language, and integrated up-to-date bioinformatics tools to improve usability, robustness and reproducibility. RNA-Seq analysis of the data from irradiated human PDAC cell line monocultures, as well as PDAC and CRC patient tumors, revealed global deregulation of autonomous ERVs, with plenty of them being strongly upregulated (Supplementary File 1). In PDAC, 26 of the autonomous ERVs induced in the PDAC cell lines are also induced in the PDACs of irradiated human patients (Fig. 5A), and their majority are within the range of 400–800 bps (Fig. 5B).

We further examined conservation of the autonomous ERVs of human irradiated tumors in mice. To identify the mouse ERVome changes, we applied the Repenrich 2.0 pipeline ^44^ to the datasets of Table 1. Similar to the patient tumors, we observed genome-wide ERVome changes in the CONV-irradiated PDAC (Fig. 5C) and CRC (Fig. 5D) mouse tumors versus their non-irradiated controls. Eighteen (18) autonomous ERVs induced either in the PDAC cell lines and/or PDAC patient tumors (Fig. 5E) were also upregulated in the irradiated mouse PDACs, while their majority are 400–800 bp-long (Fig. 5F). Therefore, we found conservation of the same 400–800-bp ERVs along with co-expressed sensors that recognize these lengths (OASes, RLRs) in mouse and human PDACs that exhibit phenotypes of RT-induced antitumor immunity. No common significantly significant autonomous ERVs were found between human and mouse CRCs (data not shown) perhaps due to usage of a platform with lower depth coverage for the RNA-Seq analysis of the CRC patient samples. These findings suggest that RT induces autonomous ERVs with the potential to both activate RNA sensors via their transcripts and to be translated into proteins that can act as neoantigens.

### Enhanced abscopal effects are associated with co-upregulation of common ERVs and OAS-mediated sensing in irradiated and non-irradiated tumor sites

**Fig. 6:**
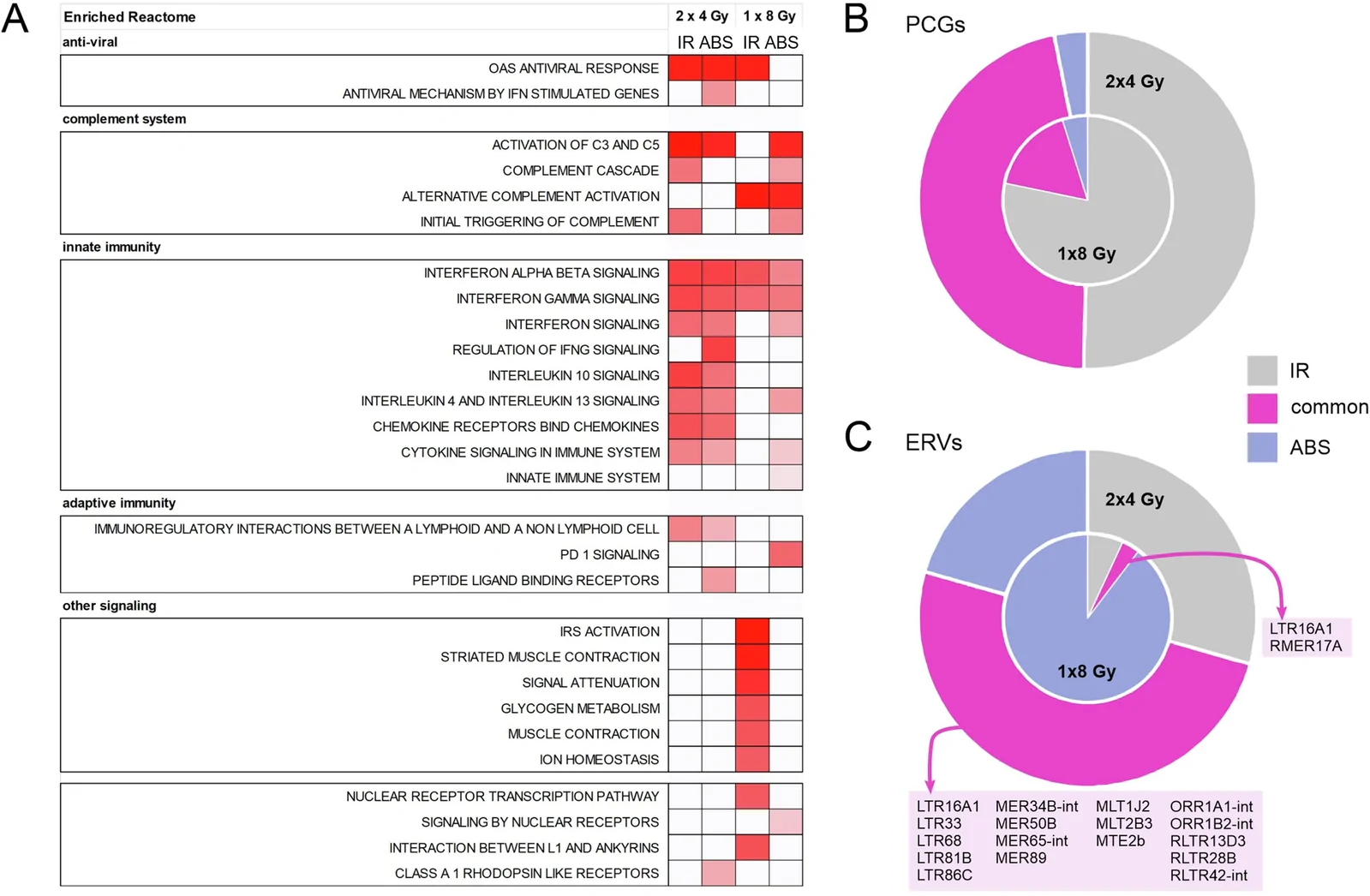
Enhanced abscopal effects are associated with transactivation of the same ERVs and viral RNA sensing in irradiated and remote sites. **A** Heatmap of overrepresented reactome processes based on the transcriptomes of lymphomas inside (IR) and outside (ABS) the irradiation field following treatment of mice with 2x4 Gy or 1×8 Gy. For the 2x4 Gy regimen, which elicited enhanced abscopal effects, OAS response is overrepresented both in IR and NIR sites. **B** Circle diagrams of upregulated PCGs in the IR and ABS tumors treated with 2x4 Gy (outer circle) versus 1×8 Gy (inner circle). Purple segments represent the percentage of common PCGs between IR and ABS tumors. In the case of 2×4 Gy, the purple segment is larger, representing greater overlap. **C** Same as (**B**) for upregulated LTR/ERVs. The common ERVs between IR and ABS for each regimen are noted.

In the lymphoma study (Table 1, GSE281695), A20 cells were injected into both sides of the mice, and two irradiation regimens were tested separately, 1x8-Gy or 2x4-Gy. One of the developed tumors (IR) received irradiation, while the other tumor remained non-irradiated (hereafter termed as abscopal tumor-ABS). Although both regimens exerted similar effects on the IR tumors, enhanced abscopal effects in the ABS tumors were reported only for the 2x4-Gy regiment, 7 days post-RT, but not for 1x8-Gy. Transcriptome analysis of the 2x4-Gy ABS tumor revealed activation of the same pathways as in the matched IR tumor, since 10 of the 12 top-enriched pathways in the IR (83.3%) were reproduced in the ABS tumor. Contrariwise, the 1x8-Gy sheme transactivated far fewer pathways in the ABS versus IR tumor (25%, 3 of the 12-top enriched in IR tumors) (Fig. 6A). Among the most enriched pathways shared between the 2x4 Gy IR and ABS tumors were the OAS-induced response to dsRNA viruses and the activation of C3 and C5 complement. Interferon pathways along with interleukin (IL0, IL4/IL13) and cytokine cascades were also recapitulated in the ABS tumor (Fig. 6A). Notably, the OAS-mediated sensing was not enriched in the 1x8-Gy ABS tumor transcriptome, despite the presence of interferon and interleukin pathways and the C3/C5 complement cascade. Moreover, half of the DEGs (116/242) detected in the 2x4 Gy IR tumors were also deregulated in the ABS tumors, compared to 17% (129/726) of the DEGs for the 1x8 Gy regimen (Fig. 6B). Therefore, the most efficient 2x4 Gy IR scheme achieved a higher reproducibility of the transcriptional changes and pathways between the IR and the ABS tumors. We further compared LTR/ERVome changes between the IR and ABS tumors. When the more efficient 2x4 Gy dose was applied, 62.9% of the LTR/ERVs activated in the IR tumors were also transcribed in the ABS tumors (Fig. 6C). In contrast, under 1x8 Gy, there were markedly fewer LTR/ERVs shared between the IR and ABS sites (16.6% versus 63%, respectively). Overall, the 2x4 Gy IR scheme promoted OAS-mediated sensing of dsRNA viruses along with transcriptional upregulation of common ERVs both in IR and ABS sites.

## Discussion

This is the first systematic correlation of organismal-level phenotypes of broad antitumor immunity with tumor transcriptional reprogramming upon exposure to homogenous or spatially heterogenous RT, to get a glimpse on the underlying mechanisms. An association between RT-induced immunogenicity and responses to RNA viruses recurrently emerged. CONV RT, at doses that establish broad antitumor immune responses in vivo, transactivates OAS/RNAse L and RIG-I pathways. In the case of microbeams, an experimental form of SFRT, OASes transcription is induced earlier along with NK cell responses. Moreover, the SFRT-induced immunogenic signatures predict patient survival more accurately than those induced by CONV RT. We also identified conserved, global changes of TE Class I transcripts, particularly LTR/ERVs, at lengths that are recognizable by OASes and RLRs. For RT regimens that elicit abscopal effects, activation of the OAS pathway and upregulation of the same LTR/ERV elements are observed in both IR and ABS sites. The co-induction of LTR/ERVs and RNA viral sensors occurs inside the tumor cells and is conserved between human and mouse.

The co-induction of OASes and RLRs with LTR/ERV RNA sequences of corresponding lengths suggests possible activation of OAS/RNAseL- and RIGI/MDA5-mediated pathways. Such pathways, which are typically triggered during a RNA viral evasion, can also take place in the absence of infections^36^. Upon dysregulation of cellular processes, vertebrate cells accumulate self-derived dsRNAs or dsRNA-like species which activate the very same immune pathways as in infected cells. The same PRRs detecting exogenous RNA viruses also sense dsRNA species, including, but not limited to, TEs that are aberrantly transcribed in a cell^36,45,46^, a reaction known as viral mimicry^47^. Viral mimicry is a cellular state in which the reactivation of silenced transposable elements (TEs) leads to the accumulation of immunogenic nucleic acids, triggering innate immune pathways that resemble responses mounted against viral pathogens. When viral mimicry is induced in a regulated setting, it triggers IFN responses and innate immunity, eventually providing immunotherapeutic benefits and tumor control^36^. By binding to RNA viral sensors, TEs repurpose the cellular defence machinery against RNA viruses to induce antitumor immune responses and combat cancer^11,48–52^. Although they were first characterized in the context of epigenetic therapies, growing evidence indicates that other cancer treatment modalities - including radiotherapy, chemotherapies, and targeted therapies - can also induce TE reactivation and viral mimicry responses in cancer cells^11^. Indeed, RT sparks ERV transcription and downstream activation of an innate antiviral MDA5/MAVS/IFN axis, thereby promoting antitumor immunity^8^.

RNA sensors, like RIG-I and MDA5, have an emerging role dsRNA-triggered viral mimicry and antitumor immunity^46^. When activated within tumors, RIG-I enhances tumor neoantigen presentation, sensitivity to immunotherapeutics and an immunoreactive TME^53–56^. Similarly, MDA5 induces apoptotic signaling and/or IFN-dependent antitumor immunity, often in co-operation with RIG-I^57^. The dsRNA-sensing pathways crosstalk with the endoplasmic reticulum stressing machinery to induce cancer cell death and interferon production^58^. Moreover, OASes, which have been long viewed as passive markers of IFN cascades, now assert active roles in the establishment of an immunoreactive TME^59^. Huai et al. demonstrated that 2–5 A, the product of the enzymatic reaction of OAS1-3, travels from donor to recipient cancer cells via gap junctions, extending the reach of RNase L-mediated innate signaling and facilitating short-range communication between cells^59^. Tumor cells with high basal OAS levels such colorectal cancer cells, naturally produce 2–5 A in vivo, which is secreted via ABCC10 to activate host-not tumor-RNAseL-mediated antitumor response. OASes are functionally related with RIG-I, since the activated RNAse L cleaves RNA to smaller fragments that serve as RIG-I ligands, eventually amplifying IRF3/IRF7-mediated signaling and interferon response^36^. Consistently, our results support strong associations between functionally intertwined RNA PPRs and RT-induced antitumor immunity, which is further indicated by the enrichment of transcripts involved in other RNA-responsive pathways, such as ISG15^28^ and/or NK signaling^60^.

Immunogenic responses to RT have traditionally been attributed to cytosolic dsDNA, which is sensed by the cGAS/STING pathway^9^. Nevertheless, RT increases IFNβ even in cGAS/STING-deficient cancer cells, indicating that cytosolic dsRNA accumulation may - alternatively or complementarily to dsDNA - activate IFN-I-mediated antitumor immunity, in certain contexts^9^. The dsRNA which is co-elevated along with dsDNA has received less attention, but nonetheless, is also immunogenic. Although the effect of the dsRNA-induced OAS-RNase L pathway on IFN production is relatively weak as compared to the most prominent cGAS/STING, it can be augmented selectively by the crosstalk between OASes and RIG-I^61^, the depletion of suppressors of the OAS-RNAseL interaction^62^, and/or overexpression of the Sp1 transcription factor^63^. For several years, cGAMP, a small molecule that triggers the dsDNA-sensing cGAS/STING pathway, has been viewed as the only known ‘immunotransmitter‘, synthesized and released by compromised cells to rapidly alert neighboring cells to a threat. In their hallmark study, Huai et al. identified 2–5 A as the second immunotransmitter, which links dsRNA-sensing pathways with tumor immunity. This study propelled the OAS/RNAseL pathway into the spotlight of anticancer targeting research^64^. In agreement, we show a recurrent relation of OAS/RNAseL with the IFN I response in irradiated tumors. These findings warrant future characterization of the downstream signaling cascades through which these RNA sensors induce in situ vaccine effects. It would also be interesting to investigate whether dsRNA-induced pathways may converge with cGAS/STING to cooperatively transactivate ISGs and potentiate immunity cascades.

Among the intratumoral TE subpopulations that are overexpressed upon immunogenic RT doses, LTR/ERVs were the most dominant, especially those at lengths recognizable by the co-upregulated RNA PRRs. ERVs are remnants of ancient retroviruses embedded in the so-called ‘junk DNA‘ regions of the human genome. They co-evolved with humans and established a complex relationship with the immune system. In normal cells, expression of ERVs remains under strict spatiotemporal control. Epigenetic silencing is the primary mechanism by which host cells keep ERV expression in-check, to maintain genomic stability. During tumorigenesis, perturbations of their delicately balanced demethylation and acetylation regulation patterns (reviewed in ref. ^65^) lead to ERV de-silencing, and their cis-activity is hijacked by oncogenes, a process known as onco-exaptation. Nevertheless, the out-of-context activation of ERVs backfires for the tumor, because their products are eventually recognized as viral threats. At the RNA level, ERV-derived transcripts activate IFN-regulated innate immune response, whilst at the protein level, ERV peptides may act as neoantigens. Considering that RT disrupts global epigenetic silencing^66^, it could act as a systematic signal to plausibly ‘awaken’ transcription of repressed ERVs^11^. If cytosolic RNA PRRs recognizing corresponding dsRNA lengths are tonically expressed and/or co-induced in cancer cells, then RNA-protein complexes could be formed inside the tumor cell, initiating viral mimicry.

Conventional homogenous RT can initiate adverse events along with immunogenic responses. In contrast, SFRT is immunogenic, but less toxic. The superiority of SFRT relies on the spatial heterogeneity of immune features^67^. Heterogenous RT leverages the distinct biological effects of low and high doses to optimize the simultaneous activation of multiple immunogenic effects in a single TME and preserve antitumor immunity. To an extent, this has been attributed to the crosstalk between the immune components of the differentially irradiated tumor volumes, which leads to tumor-infiltrating CD8+ T cells with interferon-activated phenotypes^12,19^. In the case of minibeam or microbeam irradiation, whereby the repetitive pattern of peaks and valleys generates an array of alternating high-and low-dose regions within the same tumor, the number of such favorable interactions is theoretically maximized, in proportion to the high/low-dose interfaces. In melanoma, SFRT triggers IFN I-associated antiviral pathways earlier, particularly accelerating the transactivation of OASes. It would be of interest to investigate how the temporal and spatial features mediate the superior efficacy of SFRT. An appealing hypothesis is that OASes may facilitate the paracrine transmission of innate immune signal between differentially treated compartments of the tumor. In this respect, the OAS-derived 2–5 A could be transported easier between the high-low dose-treated regions, propagating innate immune signaling and ultimately accelerating the establishment of tumor immune responses.

The simultaneous co-induction of ERVs and viral RNA sensors in the irradiated cells after immunogenic RT generates a mechanistic scenario whereby the RT-awakened ERVs interact with the RNA sensors to recruit innate immunity against the tumor via IFN pathways. These findings inform the design of future studies to characterize the molecular mechanisms of abscopal effects. Generation of study systems with selective knockout of the identified IFN-induced RNA sensors is needed to dissect the signaling cascades through which they may mediate immunoreactivity and/or RT response.

It should be emphasized that the study provides association, rather than causation, based on the publicly available RNA-Seq data and the current level of methodological advances of non-coding RNA biology. As such, it poses several limitations. First, binding of TE subpopulations to specific overexpressed innate RNA sensor(s) is poorly characterized, due to methodological limitations in RNA-protein interaction protocols, especially for the elusive class of TEs. A few studies have shown that the LTR/ERVs serve as cues of RIG-I^37,38^ and MDA5^37^, in the context of antitumor immune response, encouraging our proposed mechanistic scenario. Characterization of clinically relevant protein complexes between ERVs and RNA PRRs requires not only  sophisticated RNA-immunoprecipitation (RIP)-based assays, but also computational pipelines that accurately identify TEs in the immunoprecipitated complexes. Several RIP protocols fail to capture the RNA-protein interactions in living cells, whereas the repetitive nature and  the incomplete mapping and annotation of TEs^40^ challenge the standardization of computational pipelines that identify the protein-bound TEs with minimal background noise. Methods such as individual nucleotide resolution crosslinking and immunoprecipitation (iCLIP) can map all the RNA interactors of any RNA binding protein of interest, at single-nucleotide resolution, in living cells, with reduced unspecific binding and decreased risk for false positives and compared to other RIP protocols^68,69^. Such protocols, combined with dedicated computational pipelines for comprehensive identification of RNA ligands^70^ can be used in the future for the high-throughput identification of the immunogenic ERVs that bind to the identified overexpressed RNA sensors. In an analogous manner, the role of ERVs not only as direct ligands but also as indirect stress signals should be addressed. Additionally, development of antibodies against peptides produced by autonomous ERVs will clarify whether ERV translation products are associated with RT-induced antitumor immunity.

Second, our approach uses several measures to exclude biases from impurities of the analyzed tumor tissues from other cell types. Tumor impurity is a major confounding factor and most immune cell types are negatively correlated with tumor purity. In this regard, we excluded, from Table 1, publicly available studies where single-cell RNA-Seq analysis was performed selectively on CD45+ T cells infiltrating the irradiated tumor; we used the option ‘Purity Adjustment‘ of TIMER2.0 database, to generate the associations between gene expression and immune cell infiltration; and we experimentally validated expression of components of OAS/RNAseL and RIG-I pathways in RT-treated cancer cell monocultures. Nevertheless, we cannot completely rule out the possibility that those pathways are simultaneously activated in other cellular components of the heterogenous tumor tissue, such as immune cells, contributing in the enhancement of RNA virus sensing pathways in the available RNA-Seq data from mouse tumors. Sensitive spatial omics are essential to capture the heterogeneity and architecture of the irradiated tumors. Furthermore, mice models with knockout for selected immune cell types can further clarify the degree of contribution of tumor cells, immune cells or both in the activation of dsRNA pathways in the irradiated tumor.

Third, the associations between SFRT and tumor-intrinsic immunogenic signaling derive from microarrays on melanoma mouse tumors. Trappetti et al.^14^ has conducted the most recent and comprehensive in vivo study, incorporating more updated probeset annotations than the older glioblastoma studies. However, it is focused on one cancer type and does not provide insights on changes in the transposable elements. Therefore, more in vivo studies on the effects of SFRT on mouse models for additional cancer types are essential to clarify whether the emerged associations are melanoma-specific or have a more generalized nature. The selection of RNA-Seq rather than mRNA microarray platforms will also provide insights on putative mediators not covered by microarrays, such as transposable elements and other non-coding RNA players. The compatibility of SFRT application with the cancer type needs to be considered for the sound planning of these studies, since, to date SFRT, has shown optimal results for superficial tumors^17,18^ and has entered in the clinical study phase for metastatic melanoma and soft tissue tumors (ClinicalTrials.gov ID: NCT07062003).

Finally, the conditions under which ERV awaking is beneficial for the host, especially for promoting abscopal effects, need to be specified. This is because, at least some, ERVs act as double-edged swords favoring autoimmunity^45^, aging^71^, neurodegenerative disorders^72^ or (upon chronic expression) evolution of tumor immune evasion^73^. It is important to clarify under which epigenetic conditions these enigmatic components of the dark genome are activated, as well as which of them have bona fide immunogenic properties. Addressing such limitations will facilitate us to understand how dsRNA-mediated pathways are involved in the abscopal effect and to translate the findings into tailored personalized radioimmunotherapies.

## Methods

### Mouse studies for phenotype-transcriptome correlations and identification of human orthologues

We queried the NCBI Gene Expression Omnibus (GEO) datasets using the following combinations of keywords: (“mouse” and “in vivo tumor immune response” and “irradiation”), (“mouse” and “carbon ion” and “tumor immune response”), (“mouse model” and “irradiation” and “immunogenicity”), (“mouse” and “irradiation” and “antitumor immunity”), (“irradiation” and “antitumor immunity”), (“murine”, “irradiation”, “anti-tumor immunity”), (“spatial fractionated irradiation”). We extracted all studies where mice exhibited broad antitumor responses to RT, either alone or in combination with immunotherapy, and for which bulk RNA sequencing (RNA-seq) data from both irradiated and non-irradiated (untreated) tumors are provided (last accession date: May 31, 2026). The differentially expressed genes (DEGs) in irradiated tumors compared to non-irradiated controls were detected through RNA-Seq analysis. The human homologues of the significantly upregulated mouse genes (log2FC > 1.5, *p* value ≤  0.05) were identified as previously described^74,75^.

### Bulk RNA sequencing analysis

The SRA files were downloaded from NCBI GEO and converted into FASTQ using the Sequence Read Archive (SRA) Toolkit v.3.0.0 (available at https://github.com/ncbi/sra-tools) with the *fasterq-dump* utility. Adapters and low-quality bases were removed from the raw RNA-Seq reads using Trimmomatic and the filtered reads were then aligned to either the mouse (mm10) or human genome (hg38) using STAR v2.7.1.1. Alignment files were sorted using Samtools and transcript abundance was quantified with StringTie^76^. Samples were clustered based on their expression profiles with Principal Component Analysis (PCA) in an R environment. Differential expression analysis was performed using the DESeq (v2 1.12.3)^77^ package in R.

### TE identification from RNA-Seq data

Transcript-level TE quantification was performed using TEtranscripts (v2.2.3)^78^. A dedicated Conda virtual environment was created using Miniconda3, incorporating Python (v3.8), pysam (v0.16.0.1), and R (v4.3.0). The FASTQ files corresponding to the GSE207717, GSE185311, and GSE179351 datasets were obtained from NCBI GEO, and, for each dataset, the sorted BAM files generated as intermediate outputs from the ERVmap (v1.1) pipeline were used as input for this analysis^40^. TE annotations were derived from the GRCh38_Ensembl_rmsk_TE.gtf file and its associated hg38_rmsk_TE.gtf.ind index file, both curated and publicly released by Oliver Tam (NYU Langone Health; released on May 7, 2024).

TEtranscripts was executed in multi-mapping mode to ensure quantification of reads mapping to both genes and repetitive elements. Differential TE expression analysis between the RT-treated and untreated (control) samples was conducted using DESeq2 version 1.12.3^77^. For the GSE207717 dataset, three biological comparison groups (BxPC3, MIA-PaCa2, PANC1) were defined, each consisting of three treatment and three control samples. The GSE185311 dataset included 9 SBRT-treated and 13 untreated samples, while the GSE179351 dataset comprised 6 treatment and 13 control CRC samples.

The transcript-level quantification was complemented with TElocal (v1.1.1) for locus-specific expression analysis of transposable elements^78^. The previously established Conda environment was reused, with Python (v3.8) and pysam (v0.16.0.1), thus complying with TElocal’s software requirements. Input data consisted of the sorted BAM alignment files generated by ERVmap (v1.1)^40^. Locus-level annotations were provided by the GRCh38_Ensembl_rmsk_TE.gtf and the corresponding GRCh38_Ensembl_rmsk_TE.gtf.locInd locus index file (courtesy of Oliver Tam). TElocal was executed in multi-mapping mode, processing each sample iteratively using a SLURM job submission script. Dynamic output directories and project-specific naming conventions were created for each sample to maintain data integrity and traceability. HERVd^79^, a database of human endogenous retroviruses, was utilized for assigning each element to its corresponding class (i.e. ERV, LINE, Retroposon, Satellite etc.).

### Identification of human autonomous LTRs from transcriptomic data

To identify autonomous LTR elements, we modified a previously published Perl-scripted pipeline^40^. Specifically, we re-implemented the original Perl script in Python by integrating updated bioinformatics tools for adapter trimming, sequence alignment, multithreading, error handling and dependency management. This reimplementation improved efficiency, readability, and reproducibility while maintaining full compatibility for ERV detection and quantification using paired-end RNA sequencing data. The updated Python pipeline is available at: https://github.com/IBG-ComputationalSystemsBiology/erv-mapping-pipeline.

### Quantification of repetitive elements from mouse RNA-Seq data

RepEnrich2 was installed according to the guidelines available at https://github.com/nerettilab/RepEnrich2. The software and its dependencies were installed in a cluster computing environment, including Python 2 v2.7.9 (Foundation), Bowtie2 v2.3.5.1, Bedtools v2.29.2, Samtools v1.5, and BioPython v1.76. Repetitive element annotations were obtained from RepeatMasker^80^ and were downloaded in BED file format compatible with the *Mus musculus* genome (mm9). The annotation setup was executed using the script *RepEnrich2_setup.py*, provided within the RepEnrich2 modules. The RNA-Seq reads were aligned to the reference genome using Bowtie2. Uniquely and multi-mapping reads were filtered using Samtools and the *RepEnrich2_subset.py* script. This script was modified by adding the ’Other’ class for reads not matching any LTR in the annotation file. Reads were then assigned to repeat families to quantify their expression levels, and the resulting count tables were compiled in a single Excel file containing all biological and technical replicates. After filtering out the ’Other’ category, the identified LTRs were retrieved from the annotation file. Differential expression analysis was subsequently performed using DESeq2 v1.12.3^77^ in an R environment.

### Machine learning for identifying characteristic signatures of response to CONV or SFRT

To identify robust gene signatures, Random Forest and XGBoost models were applied to rank the most significant DEGs from each mouse study, cancer type, and delivery mode (CONV or SFRT). Predictor features included the magnitude of gene expression (log2FC) and the duration of radiation exposure (time). A weighting scheme was applied, assigning weights of 0.4 to log2FC and 0.6 to exposure time, to prioritize genes with sustained, long-lasting regulation rather than transient but extreme changes, improving in this way the biological relevance of the resulting gene signatures. Preliminary analyses indicated that this weighting strategy improved ranking robustness and interpretability compared to equal weighting or approaches placing greater emphasis on log2FC^2^. All analyses were conducted in R v4.4.0.

For Random Forest, models were constructed using the randomForest package (v4.7-1.2). The weighted features served as predictor variables, while the signature score was used as the response variable. Each model was trained using 500 trees (*ntree* = 500). Variable importance was assessed by the percentage increase in mean squared error (%IncMSE), which quantifies the contribution of each predictor to model performance. The resulting importance scores were integrated with the original weighted values to compute a final combined score, from which top-ranking genes were selected as candidate signatures. For XGBoost, regression models were built using the xgboost package (v1.7.10.1). The models were trained on the weighted features with the squared error objective function (*reg:squarederror*), with a synthetic target variable defined as the sum of the weighted log2FC and exposure time. Training was performed for 100 boosting rounds with a learning rate of 0.1 and a maximum tree depth of 6. Feature importance was calculated with the *xgb.importance* function to rank candidate signature genes.

### Survival analyses, GSEA, protein Interaction Networks and tumor infiltration analyses

Cox regression analysis of The Cancer Genome Atlas (TCGA) transcriptomics, Gene Set Enrichment Analysis (GSEA) and protein interaction network construction by STRING have been described previously^74,75^. For GSEA, the Gene Ontology (GO) terms were plotted according to their enrichment percentage. For tumor infiltration analysis, the Spearman’s ρ correlation coefficients between the expression levels of each gene and the estimated infiltrate levels of selected immune cell types in the TCGA cohorts of melanoma patients (primary and metastatic) were acquired through the Tumor Immune Estimation Resource 2.0 [TIMER2.0]^32^.

### Multilayer perceptron modeling for correlation of gene signatures with patient survivability

Using the characteristic top 10 human gene homologues of CONV RT or SFRT, we modeled the expression of the ranked genes in the tumors from the SKMC TCGA cohort to predict cancer patient survival at ≥1, 3 or 5 years after diagnosis. A classification model for each survival period was built using a multilayer perceptron (MLP), i.e. a neural network composed of an input layer, hidden layers, and an output layer, whereby each layer contains a set of neurons. For each survival period, 2 hidden layers with 50 nodes per layer were used. The input features were normalized to a range of 0 to 1 using the machine learning software platform *weka 3.8.6* (University of Waikato, New Zealand). A random test set comprising 30% of the data was selected for evaluating the performance of the model. To avoid overfitting, the training data were expanded to triple their original size by adding Gaussian noise with mean = 0 and standard deviation = 0.1; the test data remain unchanged. In the study group, the survival rates in the patient cohort for 1, 3 and 5 years were 87.46%, 50.78% and 36.99%, respectively. The network was trained for 500 epochs with a learning rate of 0.3. The model performance was evaluated using the area under the curve (AUC) of the Receiver Operating Characteristic (ROC) curve by plotting the true positive rate (TPR) and the false positive rate (FPR) across different thresholds.

### Cell cultures and irradiation treatments

Panc1,  SW620 and A375 cells were cultured in DMEM, supplemented with 10% Fetal Bovine Serum and 1% penicillin-streptomycin (Thermo Fisher Scientific). For the X-ray treatments, SW620 (10^6^ cells), Panc1 (500,000 cells) and A375 (250,000 cells) were plated in 10 cm dishes. After 24 h, when the cells reached a confluency of 60–70%, they received either 8 Gy or 12 Gy radiation dose in a MultiRad225/26 irradiator (Faxitron Biotics), using the following settings: 200 kV X-rays; 17.8 mA; 0.5 mm Cu-filter; 2.151 Gy/min, 37 cm distance from the source, and 20 cm field size. The irradiated cells were then incubated at 37 °C and 5% CO_2_.

### RNA isolation, reverse transcription and real-time PCR

After 48 h (for the 12 Gy treatments) or 72 h (for 8 Gy treatments) of irradiation, cells were harvested. Total RNA was extracted using the RNeasy kit (Qiagen) according to the manufacturer’s instructions. 1 µg total RNA per sample was reverse-transcribed using the RT2 First Strand Kit (Qiagen). For real-time PCR, the cDNAs (20 ng) were mixed in a 20 µL reaction consisting of nuclease-free water, 20 pmol of each primer pair, and 7.5 µL of Power SYBR Green PCR Master Mix (Thermo Fisher Scientific). Amplification with the appropriate primers (Supplementary Table) was performed in a StepOnePlus Real-time PCR system (Thermo Fisher Scientific) using the following conditions: 95 °C for 10 min (1 cycle); 95 °C for 15 s, primer-specific annealing temperature for 30 s, 72°C for 45 s (40 cycles). Two biological replicates, each with 3 technical replicates were conducted. Statistical analyses were conducted using a two-sided Student’s *t* test.

### Protein isolation and immunoblotting

Protein lysates were prepared from Panc1 and SW620 cells 72 h after treatment with 8 Gy and their untreated controls using RIPA lysis buffer (Thermo Scientific), as previously described^81^. The protein levels were quantified using the Pierce™ BCA Protein Assay Kit (Thermo Scientific), following manufacturer’s instructions. Immunoblots were performed as previously described^82^ on 20 µg of protein lysates using antibodies against OAS/RNAseL pathway (#60125), RIG I pathway (#8348), and STING pathway (#38866) from Cell Signaling Technology; mouse anti-human beta-tubulin (MA5-16308, Thermo Scientific) was used as loading control. Three biological replicates were conducted.

### Treatment with compound 17a and cell survival assay

Compound 17a (ref. no. HY-155583) was purchased from MedChemExpresss and diluted to DMSO following manufacturer’s instructions. Panc1 cells were seeded in a 96-well black-wall, clear-bottom plates at a density of 10,000 cells per well. 24 h later, cells were treated with compound 17a at a concentration of 4 μM. Corresponding DMSO-treated controls were prepared. After 2 h of incubation with the drug or diluent at 37 °C in 5% CO_2_, half of the plates were irradiated (8 Gy), while the other half served as untreated controls. Cell viability was assessed 24, 48 and 72 hours after irradiation, using the PrestoBlue^TM^ Cell Viability Reagent (Invitrogen). In brief, the medium was removed and the cells were washed with 100 μL of HBSS. Cell culture medium containing 10% PrestoBlue reagent was then added to each well. The plates were incubated for 2 h at 37 °C, 5% CO_2_ and fluorescence was measured using CLARIOstar plate reader at an excitation wavelength of 557-15 nm and an emission wavelength of 597-14 nm. Three independent biological replicates, with 6 technical replicates each were conducted.

## Supplementary information

41698_2026_1715_MOESM1_ESM.

## Data Availability

The transcriptomics data analyzed in this study are publicly available in the Gene Expression Omnibus (GEO) repository under the following accession numbers: GSE56113, GSE61208, GSE168016, GSE179351, GSE185311, GSE207717, GSE209765, GSE255242, GSE268311, GSE281695, GSE290674, and in the ArrayExpress repository under the number E-MTAB-13096.

## Code availability

The Python script is available at: https://github.com/IBG-ComputationalSystemsBiology/erv-mapping-pipeline.
