# Supplementary material for: Intratumoral co-activation of ERV transcription and RNA virus-sensing is associated with radiotherapy-induced immune response and abscopal effect
Source: NPJ Precis Oncol. 2026 Oct 13;10:421. doi: 10.1038/s41698-026-01715-4 (PMC13731661; doi:10.1038/s41698-026-01715-4)
Supplement: Supplementary file 1 [file 41698_2026_1715_MOESM1_ESM.docx]

**Supplementary Figure 1: Conserved CONV RT and SFRT- upregulated transcripts across cancer types.** The grey columns represent rodent genes with conserved human orthologues. The number of DEGs varied across cancer types, perhaps due to differences in cancer type, irradiation dose, and transcriptomic platforms. CONV transactivates relatively few genes in melanoma and glioblastoma, but this is reversed by SFRT, implying the ability of the latter to boost transcriptional activation.

**Supplementary Figure 2: Upregulated genes and pathways in SFRT-treated tumors and correlation with tumor immune infiltration** (A-B) Venn diagrams of human orthologues of the genes that are transcriptionally elevated in melanomas and glioblastomas two (A) or seven days (B) after SFRT (microbeam). Members of the same protein families appear in red lettering (C) Enriched reactome of the 101 shared upregulated genes in melanomas and glioblastomas 7 days after SFRT. Early common response patterns include transcriptional activation of members of the CCL and CXCL cytokine families, Toll-like receptors, and OAS proteins, all of which are involved in the recognition and/or response to pathogen-derived ligands. At later stages, 101 commonly SFRT-upregulated genes were particularly enriched for DAP12 signaling. (D-E) Spearman correlation diagrams of RNA expression of OAS1, OAS2, OAS3, and OASL with NK cell (D) and DC (E) infiltration of melanomas from human patients. (F) association of OAS2 mutation and NK cell infiltration in human melanoma patients.

**Supplementary Table 1: qPCR primer sequences**

| **Primer name** | **Sequence** |
| --- | --- |
| **OAS1 Forward** | GAAGCTGCCACCTCAGTATG |
| **OAS1 Reverse** | TTCCAAGACCGTCCGAAATC |
| **OAS2 Forward** | TGGCTCCTATGGACGGAAA |
| **OAS2 Reverse** | GATGTCACGTTGGCTTCTCT |
| **OAS3 Forward** | CCCAAGCCACAAGTCTACTC |
| **OAS3 Reverse** | TGGGCGAATGTTCACAAAGT |
| **OASL Forward** | GCTGAAGGTAGTCAAGGTGG |
| **OASL Reverse** | CTCCTGGAAGCTGTGGAAAC |
| **ISG15 Forward** | GCAGATCACCCAGAAGATCG |
| **ISG15 Reverse** | GGCCCTTGTTATTCCTCACC |
| **UBE2L6 Forward** | CCCTCAATGTGCTGGTGAAT |
| **UBE2L6 Reverse** | GGTGAACTCTTCGGCATTCT |
| **USP18 Forward** | TGGGAAACAGGTCTTGAAGC |
| **USP18 Reverse** | CAGGGAGTGGCAGATCTTTC |
| **RIG-1 Forward** | AGAGCAAGAGGTAGCAAGTG |
| **RIG-1 Reverse** | ATACTGCTTCGTCCCATGTC |
| **MDA5 Forward** | GAGGAATCAGCACGAGGAAT |
| **MDA5 Reverse** | ATCAGATGGTGGGCTTTGAC |
| **DDX60 Forward** | TGTTTGTCTGTCTGGGAACT |
| **DDX60 Reverse** | AAGCGACATTTTCCTTCCTC |
| **LPG2 Forward** | GCCTTGCAAACAGTACAACC |
| **LPG2 Reverse** | AATTTCCGGCTCAACTCAGG |
| **RPL0 Forward** | GGTCATCCAGCAGGTGTCC |
| **RPL0 Reverse** | CAGACACTGGCAACATTGC |
| **GAPDH Forward** | GCATCCTGGGCTACACTGAG |
| **GAPDH Reverse** | AAAGTGGTCGTTGAGGCA |
